# Supplementary material for: Rivaroxaban Monotherapy in Patients with Pulmonary Embolism: Off-Label vs. Labeled Therapy
Source: Life (Basel). 2022 Jul 27;12(8):1128. doi: 10.3390/life12081128 (PMC9409848; doi:10.3390/life12081128)
Supplement: Supplementary file 1 [file life-12-01128-s001.zip › life-1827536-supplementary.pdf]

Coordinator of the RIETE Registry: Manuel Monreal.

RIETE Steering Committee Members: Paolo Prandoni, Benjamin Brenner and Dominique Farge-Bancel.

RIETE National Coordinators: Raquel Barba (Spain), Pierpaolo Di Micco (Italy), Laurent Bertoletti (France), Sebastian Schellong (Germany), Inna Tzoran (Israel), Abilio Reis (Portugal), Marijan Bosevski (R. Macedonia), Henri Bounameaux (Switzerland), Radovan Malý (Czech Republic), Peter Verhamme (Belgium), Joseph A. Caprini (USA), Hanh My Bui (Vietnam).

RIETE Registry Coordinating Center: S & H Medical Science Service.

Members of the RIETE Group

SPAIN: Adarraga MD, Amado C, Arcelus JI, Ballaz A, Barba R, Barrón M, Barrón-Andrés B, Blanco-Molina A, Botella E, Cañas I, Casado I, Chasco L, Criado J, de Ancos C, del Toro J, Demelo-Rodríguez P, Díaz-Brasero AM, Díaz-Pedroche MC, Díaz-Peromingo JA, Díaz-Simón R, Escribano JC, Espósito F, Farfán-Sedano AI, Falgá C, Fernández-Capitán C, Fernández-Jiménez B, Fernández-Muixi J, Fernández-Reyes JL, Fidalgo MA, Font C, Francisco I, Galeano-Valle F, García MA, García-Bragado F, García de Herreros M, Gil-Díaz A, Gómez-Cuervo C, Gómez-Mosquera AM, González-Moreno M, Grau E, Guirado L, Gutiérrez J, Hernández-Blasco L, Jara-Palomares L, Jaras MJ, Jiménez D, Jou I, Joya MD, Lacruz B, Lainez-Justo S, Latorre A, Lecumberri R, Lima J, Lobo JL, López-De la Fuente M, López-Jiménez L, López-Meseguer M, López-Miguel P, López-Núñez JJ, López-Reyes R, López-Sáez JB, Lorente MA, Lorenzo A, Madridano O, Maestre A, Marchena PJ, Marcos M, Martín-Martos F, Martínez-Redondo I, Mellado M, Mena E, Mercado MI, Moisés J, Monreal M, Muñoz-Blanco A, Muñoz-Gamito G, Morales MV, Nieto JA, Núñez-Fernández MJ, Osorio J, Otalora S, Otero R, Paredes-Ruiz D, Parra P, Pedrajas JM, Pérez-Jacoiste MA, Peris ML, Pesce ML, Porras JA, Poyo-Molina J, Puchades R, Riera-Mestre A, Rivera-Civico F, Rivera-Gallego A, Roca M, Rosa V, Rodríguez-Cobo A, Rodríguez-Matute C, Ruiz-Giménez N, Ruiz-Ruiz J, Salgueiro G, Sancho T, Sendín V, Sigüenza P, Soler S, Suárez-Rodríguez B, Suriñach JM, Tiberio G, Torres MI, Torres-Sánchez A, Trujillo-Santos J, Uresandi F, Usandizaga E, Valle R, Varona JF, Vela L, Vela JR, Villalobos A, Villares P, **AUSTRIA:** Ay C, Nopp S, Pabinger I, **BELGIUM:** Engelen MM, Vanassche T, Verhamme P, **COLOMBIA:** Arguello JD, Montenegro AC, Roa J, **CZECH REPUBLIC**: Hirmerova J, Malý R, **FRANCE:** Accassat S, Bertoletti L, Bura-Riviere A, Catella J, Chopard R, Couturaud F, Espitia O, El Harake S, Le Mao R, Mahé I, Moustafa F, Plaisance L, Sarlon-Bartoli G, Suchon P, Versini E, **GERMANY:** Schellong S, **ISRAEL:** Braester A, Brenner B, Kenet G, Tzoran I, **IRAN:** Sadeghipour P, **ITALY:** Basaglia M, Bilora F, Bortoluzzi C, Brandolin B, Ciammaichella M, Colaizzo D, De Angelis A, Dentali F, Di Micco P, Grandone E, Imbalzano E, Merla S, Pesavento R, Prandoni P, Siniscalchi C, Tufano A, Visonà A, Vo Hong N, Zalunardo B, **LATVIA:** Rusa E, Skride A, Strautmane S, **PORTUGAL:** Fonseca S, Manuel M, Meireles J, **REPUBLIC OF MACEDONIA:** Bosevski M, Trajkova M, Zdraveska M, **SWITZERLAND**: Bounameaux H, Mazzolai L, **UNITED KINGDOM**: Aujayeb A, **USA:** Caprini JA, Weinberg I, **VIETNAM**: Bui HM.
